# Single HER2-positive tumor cells are detected in initially HER2-negative breast carcinomas using the DEPArray™–HER2-FISH workflow

**DOI:** 10.1007/s12282-022-01330-8

**Published:** 2022-01-13

**Authors:** Lisa Grüntkemeier, Aditi Khurana, Farideh Zamaniyan Bischoff, Oliver Hoffmann, Rainer Kimmig, Mathew Moore, Philip Cotter, Sabine Kasimir-Bauer

**Affiliations:** 1 Department of Gynecology and Obstetrics, University Hospital Essen, Hufelandstrasse 55, 45122 Essen, Germany; 2 Research Dx Inc., Irvine, CA USA; 3 Menarini Silicon Biosystems Inc., Huntingdon Valley, USA

**Keywords:** Early breast cancer, Circulating tumor cells, DEPArray™, HER2, HER2/neu FISH, Tumor heterogeneity

## Abstract

**Background:**

In breast cancer (BC), overexpression of HER2 on the primary tumor (PT) is determined by immunohistochemistry (IHC) or fluorescence in situ hybridization (FISH) to stratify samples as negative, equivocal and positive to identify patients (pts) for anti-HER2 therapy. CAP/ASCO guidelines recommend FISH for analyzing *HER2/neu* (*ERBB2*) gene amplification and for resolving equivocal HER2 IHC results. However, pre-analytical and analytical aspects are often confounded by sample related limitations and tumor heterogeneity and HER2 expression may differ between the PT and circulating tumor cells (CTCs), the precursors of metastasis. We used a validation cohort of BC patients to establish a new DEPArray™-PT-HER2-FISH workflow for further application in a development cohort, characterized as PT-HER2-negative but CTC-HER2/*neu*-positive, to identify patients with PT-HER2 amplified cells not detected by routine pathology.

**Methods:**

50 µm FFPE tumor curls from the validation cohort (*n* = 49) and the development cohort (*n* = 25) underwent cutting, deparaffinization and antigen retrieval followed by dissociation into a single-cell suspension. After staining for cytokeratin, vimentin, DAPI and separation via DEPArray™, single cells were processed for HER2-FISH analysis to assess the number of chromosome 17 and HER2 loci signals for comparison, either with available IHC or conventional tissue section FISH. CTC-HER2/*neu* status was determined using the AdnaTest *BreastCancer* (QIAGEN, Hilden, Germany)*.*

**Results:**

Applying CAP/ASCO guidelines for HER2 evaluation of single PT cells, the comparison of routine pathology and DEPArray™-HER2-FISH analysis resulted in a concordance rate of 81.6% (40/49 pts) in the validation cohort and 84% (21/25 pts) in the development cohort, respectively. In the latter one, 4/25 patients had single HER2-positive tumor cells with 2/25 BC patients proven to be HER2-positive, despite being HER2-negative in routine pathology. The two other patients showed an equivocal HER2 status in the DEPArray™-HER2-FISH workflow but a negative result in routine pathology. Whereas all four patients with discordant HER2 results had already died, 17/21 patients with concordant HER2 results are still alive.

**Conclusions:**

The DEPArray™ system allows pure tumor cell recovery for subsequent *HER2/neu* FISH analysis and is highly concordant with conventional pathology. For PT-HER2-negative patients, harboring HER2/*neu-*positive CTCs, this approach might allow caregivers to more effectively offer anti-HER2 treatment.

**Supplementary Information:**

The online version contains supplementary material available at 10.1007/s12282-022-01330-8.

## Introduction

Therapeutic decisions in breast cancer (BC) are, among other factors, based on the expression of the predictive markers on the primary tumor (PT): the estrogen-(ER) and progesterone receptor (PR) as well as the epidermal growth factor receptor HER2 [1]. However, when the PT has been removed, therapy is targeting single circulating tumor cells (CTCs) that have left the tumor and moved into secondary organs, preferentially the bone marrow as disseminated tumor cells (DTCs) [2]. A variety of studies have already demonstrated that HER2, ER and PR were differentially expressed between the PT and corresponding metastases [3–11] and/or CTCs [12–24]/DTCs [25–29]. This might explain why certain patients do not respond to anti-hormonal treatment and/or HER2-targeted therapy resulting in a worsening course of the disease. For HER2, two pilot studies have demonstrated that targeted anti-HER2 therapy was able to eliminate HER2-positive (pos) CTCs and DTCs in non-metastatic BC patients [30, 31]. However, some multicenter trials that aimed at investigating whether patients with HER2-pos CTCs but HER2-neg PT could benefit from HER2-targeted therapies (NSABP B47 and the Treat-CTC trial) failed to confirm the hypothesis that adjuvant trastuzumab can benefit women with HER2 non-amplified early BC [32–34]. To improve BC routine diagnostics, the use of DTCs is too invasive and for CTCs, no standard method has been defined until now to detect and characterize HER2-pos CTCs so that their clinical utility has been critically discussed [34–37]. Consequently, routine BC diagnostics of the PT, to better identify patients who could benefit from HER2-targeted therapies, has to be improved since it currently does not reveal the complex intra-tumor heterogeneity and BC tumor biology.

International guidelines emphasize the importance of reproducible, accurate and quality-controlled biomarker diagnostics. With regard to HER2 status determination, clinical guidelines and stratification of BC into HER2-negative (neg), equivocal or HER2-pos cases are defined by the American Society of Clinical Oncology (ASCO) and the College of American Pathologists (CAP) [38]. In BC diagnostics, the expression intensity of HER2 is routinely determined by immunohistochemistry (IHC) using the DAKO-Score [38]. Based on this score, only patients defined as DAKO-Score + 3 or DAKO-Score + 2 and positive fluorescence in situ hybridization (FISH) for HER2 gene amplification analysis will receive anti-HER2 treatment. However, variations in HER2 results in one sample may occur due to site of pre-analytical sampling, accurate tumor assessment, proper differentiation between ductal and invasive tumor, tissue handling and intra-tumor heterogeneity. It is currently unclear whether changes of HER2 status are mostly due to inaccurate HER2 status assessment of the PT or due to the metastatic growth of a HER2-pos subclone, initially not detected within a HER2-neg PT. On the one hand, it might be assumed that tumor cells which have left the tumor might experience phenotypic and genetic differentiation during circulation, enabling them to turn from, e.g. HER2-neg on the PT to HER2-pos on single tumor cells and/or metastases and vice versa. On the other hand, it might be suggested that a few HER2 expressing tumor cells that are already existing within the PT are leaving to give rise to HER2-pos single cells and/or metastases. However, these initially HER2-pos cells are usually not detected by routine pathology. Although the underlying mechanisms of these theories have not been resolved, it has been proposed that the clinical efficacy of HER2 blockade in tumors classified as HER2-neg might be explained by the “cancer stem cell hypothesis” where cancers, including BC, are driven by a subpopulation of cells that display stem cell properties [39]. Furthermore, HER2 was shown to be selectively expressed in the cancer stem cell population of luminal ER + BC in the absence of HER2 gene amplification and provided evidence that the efficacy of HER2 blocking agents in the adjuvant setting may reflect effects on these cells [40].

However, therapeutic decisions in the clinic are still based on the expression of predictive markers on the PT, although the use of CTCs might be more challenging, especially in monitoring studies. To address this challenge, we used FFPE tissue of 49 BC patients (validation cohort) to establish and validate a new DEPArray™-PT-HER2-FISH workflow which provides a single-cell, image-based sorting of a pure tumor cell population prior to HER2-FISH analysis. In a second step, we aimed to evaluate BC intra-tumor heterogeneity using the DEPArray™-HER2-FISH workflow in another 25 BC patients (development cohort) characterized as HER2-neg (DAKO-Score 0 or + 1) on the PT but HER2/*neu-*pos on CTCs. It was our purpose to detect HER2-amplified cells in the PT that were not detected by routine pathology to identify patients who might benefit from for anti-HER2 treatment.

## Patients and methods

### Patient characteristics

**Cohort 1:** Validation cohort for establishment of the DEPArray™-HER2-FISH workflow

For method establishment, 54 breast carcinoma samples were obtained from commercial tissue banks by Menarini Silicon Biosystems (Huntingdon Valley, PA, USA). The detailed results for all patients are documented in Suppl. Table 1. 49/54 samples met pre-analytical acceptability criteria that were also confirmed by conventional methods (IHC or Tissue FISH) to be either HER2-pos (*n* = 29) or HER2-neg (*n* = 19). One out of the 29 samples was an ERBB2-pos cell line. Another sample had no accompanying IHC or tissue FISH information, so tissue FISH was performed and the sample was classified as equivocal based on the 2013 CAP/ASCO HER2 guidelines. 21 samples were defined negative by DEPArray™-HER2-FISH workflow (including four samples that were originally positive by Tissue FISH or IHC), 22 samples were defined positive and six equivocal (two were originally negative, three were positive and one was equivocal based on Tissue FISH or IHC, respectively, resulting in an 81.6% concordance with the initial HER2 result).

**Cohort 2:** Development cohort: Assessment of HER2-amplified tumor cells in BC patients with HER2-neg primary tumors (DAKO-Score 0, + 1) and HER2/*neu*-pos CTCs was conducted at the Department of Gynecology and Obstetrics in Essen, Germany. In total, 25 primary, non-metastatic BC patients with first diagnoses between August 2007 and July 2010, were evaluated. All specimens were obtained after obtaining written informed consent prior to inclusion in the study and collected using protocols approved by the institutional review board (05/2856; 16-6915-BO).

The eligibility criteria were as follows: histologically proven BC, no severe uncontrolled co-morbidities or medical conditions, no further malignancies at present or in history, completion of adjuvant treatment according to guidelines including adjuvant chemotherapy (anthracyclines, 5-fluorouracil, taxanes, cyclophosphamide) and anti-hormonal therapy in case of hormone-responsive tumors (tamoxifen or an aromatase inhibitor). All patients had a HER2-neg PT (DAKO-Score 0, + 1) and HER2/neu-pos CTCs. Patient characteristics at the time of diagnosis are shown in Table 1. The median age of the patients was 57 years, range 31–80 years. 11/25 (44%) patients had T1 and 12/25 (48%) had T2 tumors, respectively. 15/25 (60%) were node-negative and the majority of the patients had a ductal carcinoma (19/25; 76%) and a predominantly poor or moderately differentiated tumor (19/25; 76%). ER and/or PR positivity was observed in 84% (21/25) of the tumors. Whereas 17/25 patients (68%) are still alive, 8/25 (32%) died after 2–10 years.

**Table 1:** Clinical data of the development cohort

Total	25
Median age	57 years (range 31–80 years)
Tumor size	
pT1	11
pT2	12
pT3	2
Nodal status	
Negative	15
Positive	10
Histology	
Ductal	19
Lobular	5
Other	1
Grading	
I	6
II	15
III	4
ER status^1^	
Negative	4
Positive	21
PR status^1^	
Negative	4
Positive	21
HER2 status^1^	
Negative	25
Positive	0
Menopausal status	
Premenopause	3
Perimenopause	5
Postmenopause	17

^1^Determined by IHC

### Selection, detection and evaluation of CTCs

Two 5-ml EDTA blood samples were collected for the isolation of CTCs before the initiation of therapy and before surgery with an S-Monovette (Sarstedt AG & Co.) and stored at 4 °C until further analysis. The samples were processed immediately or, at latest, 4 hours after blood withdrawal. CTCs were analyzed with the AdnaTest *BreastCancer* assay (QIAGEN, Hilden, Germany). Establishment and validation of this assay has been described in detail elsewhere [41]. Briefly, all samples underwent immunomagnetic enrichment targeting EpCAM and MUC1 using the AdnaTest *BreastCancerSelect* assay followed by mRNA isolation from lysed, enriched cells and subsequent reverse transcription, resulting in cDNA, which was the template for tumor cell detection and characterization by multiplex RT-PCR using the *AdnaTest BreastCancerDetect* [EpCAM, MUC-1, HER2]. Actin was used as internal PCR positive control. The primers generate fragments of the following sizes: GA 733-2: 395 base pairs (bp), MUC1: 293 bp, HER2: 270 bp and actin: 114 bp. Visualization of the PCR fragments was carried out with a 2100 Bioanalyzer using the DNA 1000 LabChips (Agilent Technologies) and the Expert Software Package (version B.02.03.SI307, both Böblingen, Germany).

### Evaluation of data

The test is considered positive if a PCR fragment of at least one tumor-associated transcript [MUC-1, GA 773–2 or HER2] is clearly detected. Using the software package for evaluation of the data on the Agilent 2100 Bioanalyzer, peaks with a concentration of > 0.15 ng/µl are positive for the transcripts GA733-2, MUC1 and HER2.

### Immunohistochemical analysis of the primary tumor

All tumor samples were analyzed for HER2 status according to the HER2 guidelines in the respective years by the pathologists [38, 42]. For patients in the development group, also the tumor type, TNM-staging and grading were assessed.

### DEPArray™-HER2-FISH workflow

The following methodology was established and validated through the analysis of a total of 54 breast cancer samples in the validation cohort. Subsequently, this method was applied to the development group of 25 patients characterized as HER2-neg (DAKO-Score 0 or + 1) on the PT but HER2/*neu*-pos on CTCs.

### Isolation and staining of single tumor cells from FFPE tissue

50 μm FFPE curls from each tumor sample were deparaffinized, rehydrated and underwent heat-induced antigen retrieval. The connected tissue was dissociated into a single-cell suspension using dispase (Life Technologies, Carlsbad, CA, USA) and collagenase (Sigma Aldrich, St. Luis, MO, USA) for enzymatic tissue degradation. The single cells were stained using two different cytokeratin (CK) 168 antibodies [clone: MNF116 (Dako/Agilent, Santa Clara, CA, USA) and clone: AE1/AE3 (Merck 169 Millipore, Burlington, MA; USA)] and one vimentin (Vim) antibody [clone: 3B4 (Dako/Agilent, Santa 170 Clara, CA, USA)]. Primary antibody binding of CK was visualized by AF488 secondary antibody (Life Technologies, Carlsbad, CA, USA), Vim binding was visualized by AF647 (Life Technologies, Carlsbad, CA, USA). DAPI (Sigma Aldrich, St. Luis, MO, USA) served as nuclear staining.

### DEPArray™ run

Subsequently, the stained single-cell suspension was prepared as recommended by the manufacturer and 13 μl of the single-cell suspension containing a maximum 30.000 cells was loaded into the cartridge and processed on the DEPArray™ (Menarini Silicon Biosystems, Huntingdon Valley, PA; USA). After scanning the main chamber for the presence of single cells, the gating strategy (Suppl. Fig. 1) adoapted for target cell identification was as follows: from all cells present in the main chamber, only those being trapped in the cage and, therefore, movable in the cartridge, were selected. In a scatterplot based on cells being in cage, CK-AF488 and Vim-AF647 were displayed and the two populations, CK-pos/Vim-neg/DAPI-pos tumor cells and Vim-pos/CK-neg/DAPI-pos stroma cells, were defined. The DNA index of the two cell populations was defined by using the integral intensity DAPI. The Vim-pos/CK-neg/DAPI-pos diploid stroma cells served as normal DNA reference to identify the diploid and hyperploid tumor cell fractions. Samples containing at least 100 viable CK-pos/Vim-neg/DAPI-pos tumor cells were deemed suitable for tumor cell recovery and subsequent HER2-FISH.

### Evaluation of HER2-amplified cells by FISH

After pure tumor cell recovery, 5–10 μl of sample remaining after volume reduction was spot dropped on a pre-labeled and pre-cleaned positively charged slide and etched on the backside to mark the spread of cells. Slides were baked at 65 °C in a hot air oven for a minimum of 15 min and maximum of only 30 min. Baked slides were rehydrated by 1-min washes each in 100%, 85% and 70% ethanol. Slides were then immersed in Pepsin solution preheated to 37 °C for one minute and agitated for uniform digestion. After the excess, pepsin was drained and slides were dehydrated in ascending alcohols of 70%, 85% and 100% for 2 minutes, each followed by air drying and probe application. The dual ERBB2/CC17 probe (Biocare Medical, Pacheco, CA, USA) was applied and sealed with a coverslip. The slide was denatured at 72 °C for 2 min., followed by hybridization for 12 to 24 h at 37 °C on the Thermobrite. The next day, the slide was washed twice in post-hybridization buffer at room temperature and at 72 °C. The slide was air dried and DAPI was applied for nuclear staining.

Up to 100 nuclei were evaluated and for each nucleus, the number of ERBB2 and CC17 signals was counted. The single cells, obtained after applying the DEPArray™-HER2-FISH workflow, were evaluated in two ways.

HER2 status was determined based on applying the CAP/ASCP 2013 HER2 Test recommendations (traditionally used on tissue FISH samples) to the total number of single cells analyzed on the slide. According to Wolff et al., the HER2 status was then defined as follows: HER2-pos: ERBB2/CC17 ratio > 2.2 or in average more than six copies of ERBB2 per nucleus; HER2-equivocal: ERBB2/CC17 ratio 1.8–2.2 or in average four to six copies of ERBB2 per nucleus and HER2-neg: ERBB2/CC17 ratio < 2.2 or less than four copies of ERBB2 per nucleus, respectively [38]. HER2 genetic heterogeneity in BC tumors was defined as > 5%, but < 50% of ERBB2/CC17 ratio above 2.2 in HER2 analysis [43].

## Results

### Validation of the DEPArray™—HER2 FISH workflow

#### Validation cohort

49/54 samples met pre-analytical acceptability criteria that were also confirmed by conventional methods (IHC or Tissue FISH) to be either HER2-pos (*n* = 29) or HER2-neg (*n* = 19). One out of the 29 samples was an ERBB2-pos cell line. Another sample had no accompanying IHC or Tissue FISH information, so tissue FISH was performed and the sample was classified as equivocal based on the 2013 CAP/ASCO HER2 guidelines.

**Fig. 1:**
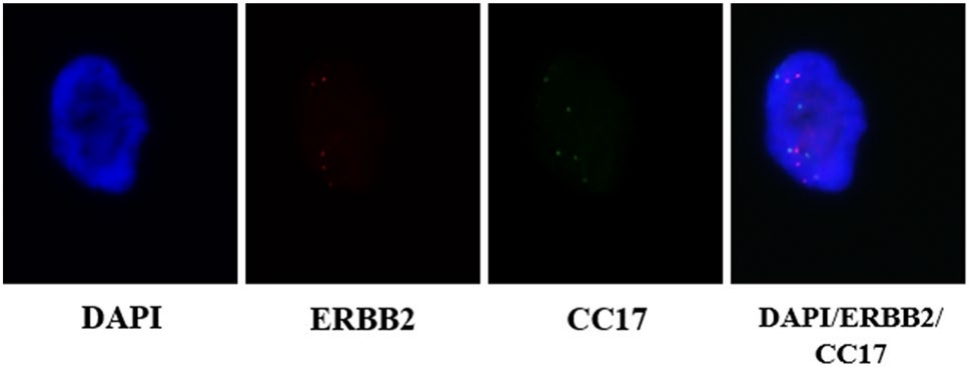
Exemplarily DEPArray™-HER2-FISH image for one sample from the validation cohort. Image shows single channels and overlays of DAPI (blue)/CC17 (green)/ERBB2 (red) and were taken at 63 × magnification

Applying CAP/ASCO scoring criteria to single cells recovered after the application of the DEPArray™- HER2-FISH workflow, the following results were obtained. 21 samples were defined negative (including four samples that were originally positive by Tissue FISH or IHC), 22 samples were defined positive and six equivocal (two were originally negative, three were positive and one was equivocal based on Tissue FISH or IHC, respectively, resulting in an 81.6% concordance with the initial FISH result. In addition, the instrument performance in terms of reproducibility and reliability was reported as 100%. Figure 1 shows exemplarily a HER2-pos result.

#### Development cohort

**Fig. 2:**
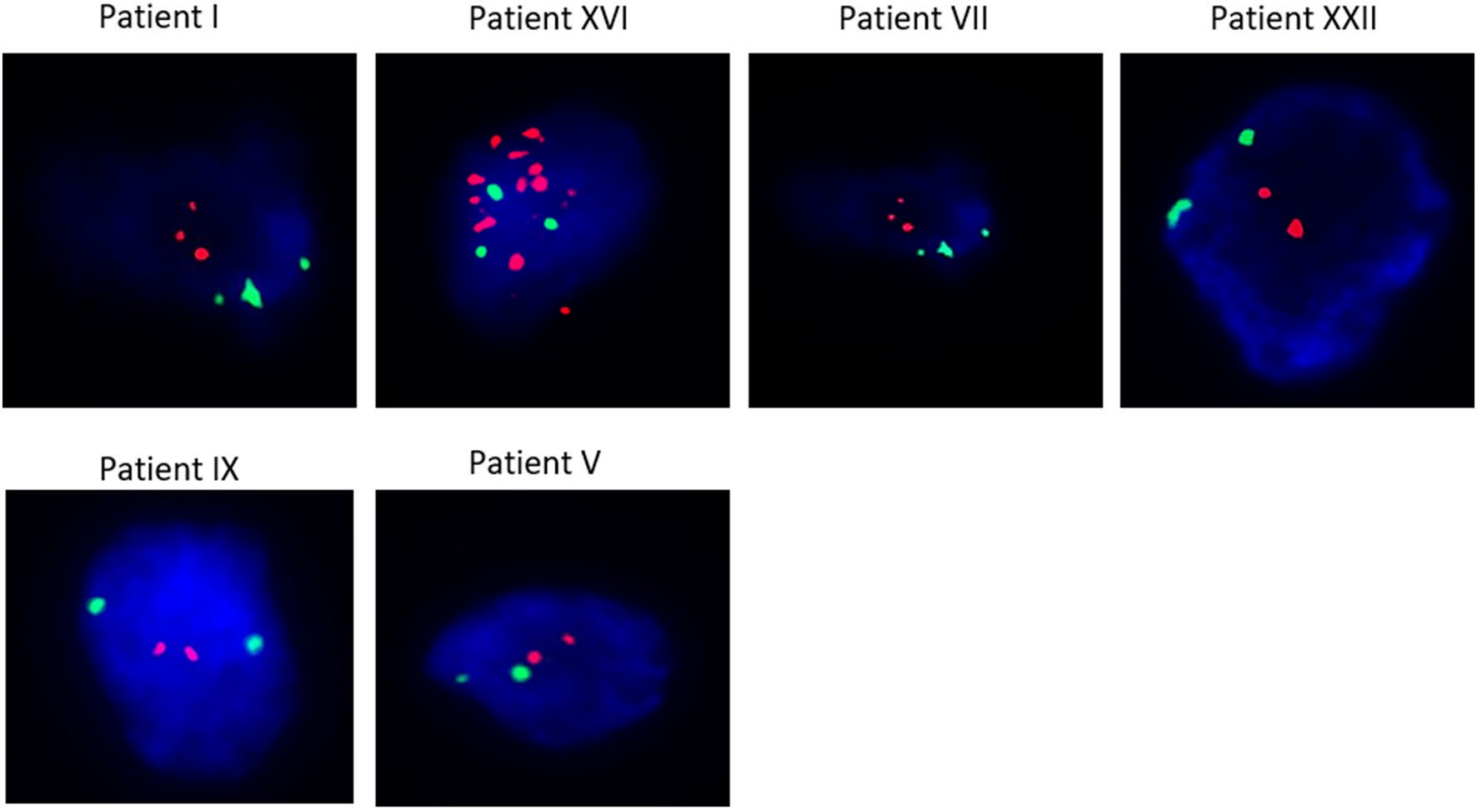
DEPArray™-HER2-FISH images for six patients being PT-HER2-neg/CTC-HER2/neu-pos. Images show overlays of DAPI (blue)/CC17 (green)/ERBB2 (red) and were taken at 63 × magnification

After successful method evaluation, 25 PT HER2-neg but CTC-HER2/*neu*-pos BC cases were analyzed for the identification of single HER2-amplified tumor cells. Whereas a concordance rate was obtained in 84% (21/25) of cases, a discordant result was found for four patients (16%). The detailed results are shown in Table 2 and exemplarily shown in Fig. 2. 2/25 BC patients (Patient I and XVI) were identified as HER2-pos by the DEPArray™-HER2-FISH workflow.

**Table 2:** Evaluation of DEPArray™-HER2-FISH results of the development cohort

Evaluation DEPArray™- HER2 FISH results				
Patient	CAP/ASCO			
	Mean ERBB2 signals	Mean CC17 signals	ERBB2/CC17 ratio	HER2 status
I	6.01	3.95	1.52	pos
II	1.92	1.30	1.48	neg
III	2.47	2.03	1.22	neg
IV	2.17	2	1.08	neg
V	2	2	1	neg
VI	2	2	1	neg
VII	5.16	4.18	1.24	equi
VIII	2.42	2.33	1.04	neg
IX	1.83	1.83	1	neg
X	2.27	2.0	1.14	neg
XI	1.83	1.83	1	neg
XII	2.62	1.99	1.32	neg
XIII	2.2	2.07	1.07	neg
XIV	2.02	2	1.01	neg
XV	2.67	2.19	1.22	neg
XVI	9.5	3.25	2.92	pos
XVII	2	1.71	1.17	neg
XVIII	3.08	2.58	1.19	neg
XIX	2	2	1	neg
XX	1.88	1.82	1.03	neg
XXI	2.23	2.01	1.11	neg
XXII	4	2.54	1.57	equi
XXIII	2.66	1.95	1.37	neg
XXIV	2.48	2.12	1.17	neg
XV	2.3	2.14	1.08	neg

DEPArray™-HER2-FISH results from discordant HER2 BC cases defined as PT-HER2-negative by routine pathology, but CTC-HER2-positive

*equi* equivocal, *neg* negative, *pos* positive

In detail, patient XVI was defined as HER2-pos by an ERBB2/CC17 ratio > 2.2 while also having more than six ERBB2 copies per nucleus. Patient I showed more than six ERBB2 copies per nucleus, whereas no positive ERBB2/CC17 ratio was detected. Two other patients (Patient VII and XXII) were found to be equivocal by both, having four to six ERBB2 gene copies per nucleus (Fig. 2 and Table 2). 12/25 (48%) patients had at least one detected single HER2-amplified tumor cell using the new methodology.

Remarkably, all four patients (patients I, XVI, VII and XXII) with discordant HER2 results between pathology and DEPArray™-HER2-FISH died after 3–11 years after first diagnosis and only 4/21 patients who showed concordant HER2-neg results died within the same follow-up period.

## Discussion

Despite international recommendations for HER2 testing defined by CAP/ASCO [38], the results of HER2 analyses can vary due to pre-analytical sampling, tissue handling and intra-tumor heterogeneity. CTC-based HER2 status assessment became of primary interest 10 years ago when Meng et al. reported that ERBB2-amplified CTCs were detected at the time of tumor progression in HER2-neg metastatic BC patients [44]. Following this publication, discordance between HER2 expression on the PT in comparison with metastases and/or single tumor cells in blood and bone marrow has been widely demonstrated [45]. The mechanism behind this phenomenon remains unknown; however, it may represent a cause of significant biological and therapeutical consequences. Therefore, the new DEPArray™-HER2-FISH workflow was established to potentially overcome HER2 discordance and to identify single HER2-pos tumor cells in HER2-neg PT tissue.

We demonstrated high concordance between routine pathology HER2 testing and DEPArray™-HER2-FISH analysis in both, the validation and development cohort, evaluating according to routine CAP/ASCO guidelines. However, in the development cohort, four BC patients were identified as HER2-pos/equivocal with initially HER2-neg PTs, but HER2/*neu*-pos CTCs. Slightly lower concordance rates were described between conventional HER2-IHC and FISH: (I) FISH and HER2 + 2 from 17 to 72% and (II) FISH and HER2 + 3 from 51 to 100% [46–51].

This phenomenon was also observed by others, although at lower frequencies [51, 52] and may not be caused by polysomy of chromosome 17 [53]. The false-negative rate for the HercepTest in HER2-FISH-pos BC cases was reported in 28% of cases [52]. False-positive results have also been reported by others [46, 52], but did not occur in our validation. Numerical aberrations of chromosome 17 have been designated to be the major cause of HER2-equivocal test results [54] and were present in 65% of HER2- amplified cases [55]. Another explanation for HER2 discordance is spatial tumor heterogeneity with regard to HER2 expression in tumor tissue [56]. For the validation cohort, where tissue was purchased from a commercial tissue bank, it is unknown whether the initial pathology results were obtained from the same FFPE sample block, another tumor block, or from a tumor biopsy as discrepancies of over 20% can occur between whole tumor and biopsy HER2 analysis [57].

Contrary to Wojnar et al. we did not find any discrepancies between HER2 status of the biopsy (pathology) and whole tumor (DEPArray™-HER2-FISH) in the development cohort. However, 4/25 (16%) patients defined as PT-HER2-neg but harboring HER2/*neu*-pos CTCs showed a discordant HER2 status: two were classified as HER2-pos and two as HER2-equivocal. Consequently, the two newly diagnosed HER2-pos patients would probably have benefited from anti-HER2 treatment. According to the current guidelines, there is lacking evidence whether HER2-equivocal BC patients benefit from anti-HER2 treatment [38]. Nevertheless, we propose that HER2-equivocal BC patients with HER2/*neu*-pos-CTCs could benefit from anti-HER2 treatment as BC therapeutics are per se designated to treat minimal residual disease reflected by CTCs. Thus, the inclusion of HER2 testing on CTCs into clinical guidelines might be a valuable approach; however, analytical difficulties might occur since HER2 genetic heterogeneity within the PT has been observed in 5% to 40% of BC cases [58, 59] which also confirms the detected rate in our cohort.

One aim of analyzing BC patients with PT-HER2-neg but CTC-HER2/*neu*-pos characteristics was to investigate the hypothesis that a very few HER2-amplified cells do exist in the PT but are not detected by routine pathology and might explain the discrepancies in HER2 expression. We detected at least one single HER2-amplified cell in 12/25 (48%) of these patients, which supports this theory. However, the occurrence of genetic changes during the process of tumor cell dissemination is still plausible due to clonal evolution of tumor cells [56]. Especially after leaving the primary tumor, there is a need to adapt and survive in the new microenvironment blood. Tumor cells have a certain degree of genetic instability and experience genetic mutations during tumor development and progression, leading to different tumor cell characteristics in one tumor as well as to differences in metastasis or minimal residual disease [60].

CAP and ASCO have well-defined guidelines and stratification of BC into HER2-neg, equivocal or HER2-pos cases and our concordance rate of more than 80% in the validation cohort comparing routine pathology and DEPArray™-HER2-FISH analysis strengthens this definition. Furthermore, in daily clinical routine, such a comprehensive analysis by using the DEPArray™-HER2-FISH analysis is not feasible for a large patient numbers and would be extremely cost-ineffective. However, subgroups of BC patients, in this context, the subgroup of triple-negative BC (TNBC) patients, accounting for 15% of BC cases and who do not receive anti-hormonal or anti-HER2 treatment, might be an interesting target population. In this context, it was demonstrated that CTCs of early stage TNBC patients frequently expressed ER, PR, HER2 and the epidermal growth factor receptor (EGFR) with a predomination of the latter one over the other phenotypes [61]. Very recently, using a 17-gene panel for the comprehensive characterization of CTCs in TNBC patients, we demonstrated that EGFR-pos/ERBB2-pos/ERBB3-pos CTCs before therapy as well as ERBB2-pos/ERBB3-pos CTCs after therapy were a strong predictor for a reduced PFS, with a dominating influence of EGFR and ERBB3 before but ERBB2 and ERBB3 after therapy [62]. Thus, this patient group might be in the focus of a more precise HER2 analysis to treat them accordingly, especially when also HER2-pos CTCs are detected.

Several clinical studies have been conducted and are still ongoing to treat primary and metastatic BC patients according to the presence of HER2-pos CTCs and or DTCs [30–34]. Whereas two studies in primary BC demonstrated successful single-cell tumor elimination in blood and bone marrow by anti-HER2 treatment [30, 31], the NSABP B47 and the Treat-CTC trial failed to confirm the hypothesis that adjuvant trastuzumab can benefit women with HER2 non-amplified early BC [33, 34]. In the metastatic setting, clinical studies reported a limited success targeting HER2-pos CTCs [32, 34]. Thus, optimal treatment options for BC patients who had HER2-neg primary tumors but positive HER2-pos CTCs are uncertain in the adjuvant as well as in the metastatic setting. In most of the studies, only the HER2-status on CTCs was evaluated without addressing other CTC characteristics. In this context, we were able to demonstrate that genes associated with resistance were frequently expressed before and after the given therapy in different BC subtypes and might have been dominating in the course of the disease [62]. Nevertheless, some interesting clinical studies are still ongoing and their results are awaited to have a more precise definition who to treat with anti-HER2-treatment, usually not eligible for such an approach. In this context, the DETECT III study, which randomizes patients with HER2-neg metastatic BC and detectable HER2-pos CTCs to standard treatment or to standard treatment in combination with lapatinib, might be of interest [63]. Preliminary results of this study look promising. Briefly, although CTC-clearance at baseline and at first follow-up did not significantly differ in the two study arms, the CTC-clearance rate was significantly associated with a longer overall survival. Interestingly, HER2-directed therapy with lapatinib had a positive impact on overall survival in these patients as compared to standard therapy alone. However, including lapatinib in the treatment protocol was not significantly associated with a stronger reduction in CTC counts as well as reduction in HER2-positive CTCs [63].

Nevertheless, HER2 analysis of primary, recurrent and metastatic tumor tissue is recommended for therapy decisions and the DEPArray™-HER2-FISH workflow could be implemented for a subgroup of BC patients like TNBC patients. From a methodological perspective, the DEPArray™-HER2-FISH workflow has the advantage to use 50 μm FFPE samples as compared to routine pathology which requires four μm-thick sections. Furthermore, the higher thickness, and, therefore, the higher number of target cells permit the image-based single cell sorting, which allows a more accurate and precise HER2 gene amplification analysis on intact and pure tumor cells. The 50-μm sample also mirrors a bigger area from the tumor tissue as compared to the four μm-slides used by the pathologists. As a disadvantage, the DEPArray™- HER2-FISH workflow requires a lengthier period of hands-on operation and is more cost intensive as compared to standard procedures. However, intra-tumor heterogeneity will not be fully elucidated by both methods.

## Conclusion

The current study is a proof of principle study to emphasize that the described protocol for the enrichment of a pure tumor cell population from FFPE samples using the DEPArray™ technology prior to subsequent single cell analysis, in our setting HER2-FISH analysis, is a reliable and reproducible method. This method evaluation supports the understanding that tumor heterogeneity can result in discordant results effecting BC patient treatment. Single HER2-amplified tumor cells were detected in initially HER2-neg BC patients having HER2/neu-pos CTCs. This supports the assumption that single HER2-amplified cells do exist at primary diagnosis but stay undetected by conventional HER2 analysis. Although not feasible for the entire group of BC patients in daily routine, the inclusion of more comprehensive HER2 diagnostic might be valuable approach with regard to treatment decisions in a subgroup of patients. Despite HER2-FISH analysis, the use of the DEPArray™ system is a powerful tool for investigating tumor heterogeneity for phenotypic and genotypic characterization of single tumor cells expressing other therapeutic targets of interest.

## Supplementary Information

Below is the link to the electronic supplementary material.

Supplementary file1 Suppl Figure 1: Gating strategy for target cell identification. From all cells present in the main chamber, only those being trapped in the cage and, therefore, movable in the cartridge, were selected. In a scatterplot based on cells being in cage, CK-AF488 and Vim-AF647 were displayed and the two populations, CK-pos/Vim-neg/DAPI-pos tumor cells and Vim-pos/CK-neg/DAPI-pos stroma cells, were defined. The DNA index of the two cell populations was defined by using the integral intensity DAPI. The Vim-pos/CK-neg/DAPI-pos diploid stroma cells served as normal DNA reference to identify the diploid and hyperploid tumor cell fractions. Samples containing at least 100 viable CK-pos/Vim-neg/DAPI-pos tumor cells were deemed suitable for tumor cell recovery and subsequent HER2-FISH (PNG 193 KB)

Supplementary file2 (DOCX 25 KB)

## Data Availability

The datasets used and/or analyzed during the current study are available from the corresponding author on reasonable request.

## Abbreviations

BC: Breast cancer
CC17: Copy control 17
CK: Cytokeratin
CN: Copy number
CTC: Circulating tumor cell
EpCAM: Epithelial cell adhesion molecule
Equi: Equivocal
ER: Estrogen receptor
ERBB2: Gene encoding for the human epidermal growth factor receptor 2
FFPE: Formalin-fixed paraffin-embedded
FISH: Fluorescence in situ hybridization
HER2: Human epidermal growth factor receptor 2
IHC: Immunohistochemistry
MUC-1: Mucin-1
neg: Negative
pos: Positive
PR: Progesterone receptor
PT: Primary tumor
pts: Patients
VIM: Vimentin
