# Supplementary figures and images for: Single HER2-positive tumor cells are detected in initially HER2-negative breast carcinomas using the DEPArray™–HER2-FISH workflow
Source: Breast Cancer. 2022 Jan 13;29(3):487–97. doi: 10.1007/s12282-022-01330-8 (PMC9021056; doi:10.1007/s12282-022-01330-8)

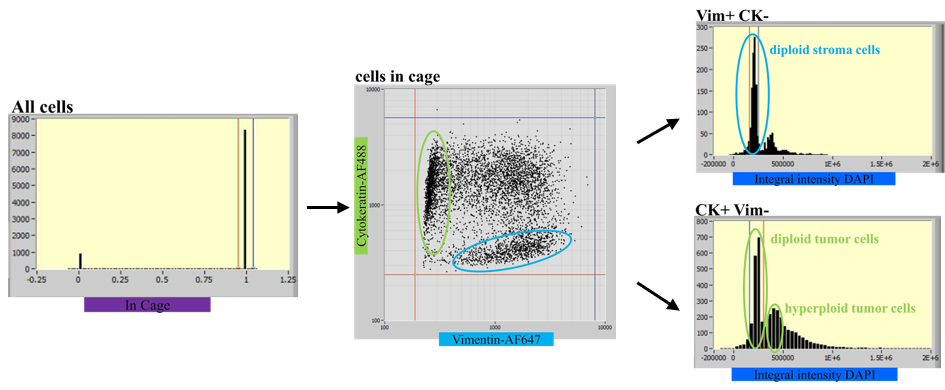

Supplement: Supplementary file 1 — Supplementary file1 Suppl Figure 1: Gating strategy for target cell identification. From all cells present in the main chamber, only those being trapped in the cage and, therefore, movable in the cartridge, were selected. In a scatterplot based on cells being in cage, CK-AF488 and Vim-AF647 were displayed and the two populations, CK-pos/Vim-neg/DAPI-pos tumor cells and Vim-pos/CK-neg/DAPI-pos stroma cells, were defined. The DNA index of the two cell populations was defined by using the integral intensity DAPI. The Vim-pos/CK-neg/DAPI-pos diploid stroma cells served as normal DNA reference to identify the diploid and hyperploid tumor cell fractions. Samples containing at least 100 viable CK-pos/Vim-neg/DAPI-pos tumor cells were deemed suitable for tumor cell recovery and subsequent HER2-FISH (PNG 193 KB) [file 12282_2022_1330_MOESM1_ESM.png]
