# Supplementary material for: Single HER2-positive tumor cells are detected in initially HER2-negative breast carcinomas using the DEPArray™–HER2-FISH workflow
Source: Breast Cancer. 2022 Jan 13;29(3):487–97. doi: 10.1007/s12282-022-01330-8 (PMC9021056; doi:10.1007/s12282-022-01330-8)
Supplement: Supplementary file 2 — Supplementary file2 (DOCX 25 KB) [file 12282_2022_1330_MOESM2_ESM.docx]

|  | **HER2 Tissue results*** | | | **DEPArray HER2-FISH results**** | |  |
| --- | --- | --- | --- | --- | --- | --- |
| **Sample number** | **IHC**  **Dako Score** | **HER2 FISH ratio** | **HER2 status** | **Average ERBB2 copy number*** | **HER2 status** | **Concordance?** |
| **1** | / | 1 | negative | 2,26 | negative | yes |
| **2** | / | 1 | negative | 1,83 | negative | yes |
| **3** | / | 1 | negative | 2,43 | negative | yes |
| **4** | / | 1,03 | negative | 2,29 | negative | yes |
| **5** | / | 1,59 | negative | 5 | equivocal | no |
| **6** | / | 1,69*** | equivocal | 4,91 | equivocal | yes |
| **7** | 0 | / | negative | 2,4 | negative | yes |
| **8** | 0 | / | negative | 5,2 | equivocal | no |
| **9** | 0 | / | negative | 2,3 | negative | yes |
| **10** | 0 | / | negative | 3,5 | negative | yes |
| **11** | 0 | / | negative | 2,5 | negative | yes |
| **12** | / | 2,1**** | positive | 10,87 | positive | yes |
| **13** | + | / | negative | 2 | negative | yes |
| **14** | + | / | negative | 1,98 | negative | yes |
| **15** | + | / | negative | 2,56 | negative | yes |
| **16** | +++ | / | positive | 7 | positive | yes |
| **17** | +++ | / | positive | 3,62 | negative | no |
| **18** | / | 2,1 | positive | 9,25 | positive | yes |
| **19** | / | 2,2 | positive | 5,18 | equivocal | no |
| **20** | / | 2,2 | positive | 4,2 | equivocal | no |
| **21** | / | 5,8 | positive | 10,62 | positive | yes |
| **22** | / | 6,34 | positive | 2,4 | negative | no |
| **23** | +++ | / | positive | 7,5 | positive | yes |
| **24** | / | 1,2 | negative | 2 | negative | yes |
| **25** | +++ | / | positive | 9,36 | positive | yes |
| **26** | +++ | / | positive | 8,97 | positive | yes |
| **27** | / | 1,6 | negative | 3 | negative | yes |
| **28** | +++ | / | positive | 8,19 | positive | yes |
| **29** | +++ | / | positive | 6,7 | positive | yes |
| **30** | +++ | / | positive | 7,43 | positive | yes |
| **31** | +++ | / | positive | 4,2 | equivocal | no |
| **32** | +++ | / | positive | 3,44 | negative | no |
| **33** | / | 8,68 | positive | 6,8 | positive | yes |
| **34** | / | 10,7 | positive | 10,82 | positive | yes |
| **35** | / | 10,96 | positive | 9,74 | positive | yes |
| **36** | / | 11,82 | positive | 2,15 | negative | no |
| **37** | / | 17,21 | positive | 8,33 | positive | yes |
| **38** | +++ | / | positive | 14,12 | positive | yes |
| **39** | +++ | / | positive | 6,1 | positive | yes |
| **40** | +++ | / | positive | 8,71 | positive | yes |
| **41** | +++ | / | positive | 16 | positive | yes |
| **42** | +++ | / | positive | 6,3 | positive | yes |
| **43** | +++ | / | positive | 12,13 | positive | yes |
| **44** | +++ | / | positive | 23,46 | positive | yes |
| **45** | +++ | / | positive | 18,44 | positive | yes |
| **46** | 0 | / | negative | 2 | negative | yes |
| **47** | 0 | / | negative | 2,7 | negative | yes |
| **48** | + | / | negative | 2,1 | negative | yes |
| **49** | 0 | / | negative | 2 | negative | yes |

**Supplementary Table 1. Description of the validation cohort.**

* For HER2 tissue analysis, either IHC or FISH results were available and evaluated according to 2013 CAP/ASCO guidelines (Wolff et al., 2013).

** In DEPArray HER2-FISH workflow, only keratin-positve tumor cells were analyzed. Therefore, only the average ERBB2 copy number is available and can be evaluated according to 2013 CAP/ASCO guidelines (Wolff et al., 2013).

*** This sample had in average 4-6 ERBB2 copies per cell.
